# Matching safety to access: global actors and pharmacogovernance in Kenya- a case study

**DOI:** 10.1186/s12992-017-0232-x

**Published:** 2017-03-23

**Authors:** Kathy Moscou, Jillian C. Kohler

**Affiliations:** 1 Leslie Dan Faculty of Pharmacy, University of Toronto, 144 College Street, Toronto, ON M5S 3M2 Canada; 2 Munk School of Global Affairs1 Devonshire Place (At Trinity College), Toronto, ON M5S 3K7 Canada; 3 Dalla Lana School of Public Health, University of Toronto, 155 College Street, Toronto, ON M5T 3M7 Canada; 4 WHO Collaborating Centre for Governance, Accountability and Transparency for the Pharmaceutical Sector, University of Toronto, 144 College Street, Toronto, ON M5S 3M2 Canada; 5 School of Health Policy and Management, Faculty of Health, York University, 4700 Keele St., Toronto, ON M3J 1P3 Canada

**Keywords:** Drug safety, Global actors, Governance, Kenya, Pharmacovigilance, Regulation

## Abstract

**Background:**

The Kenyan government has sought to address inadequacies in its National Pharmaceutical Policy and the Pharmacy and Poisons Board’s (PPB) medicines governance by engaging with global actors (e.g. the World Health Organization). Policy actors have influenced the way pharmacovigilance is defined, how challenges are understood and which norms are requisite to address drug safety issues. In this paper, we investigate the relationship between specific modes of engagement among global (exogenous) and domestic actors at the national and sub-national level to identify the positive or negative effect on pharmacovigilance and pharmacogovernance in Kenya. Pharmacogovernance is defined as the manner in which governing structures; policy instruments; institutional authority (e.g., ability to act, implement and enforce norms, policies and processes) and resources are managed to promote societal interests for patient safety and protection from adverse drug reactions (ADRs). Qualitative research methods that included key informant interviews and document analysis, were employed to investigate the relationship between global actors’ patterns of engagement with national actors and pharmacogovernance in Kenya.

**Results:**

Global actors’ influence on pharmacogovernance and pharmacovigilance priorities in Kenya (e.g., legislation and adverse drug reaction surveillance) was positively perceived by key informants. We found that global actors’ engagement with state actors produced positive and negative outcomes. Engagement with the PPB and Ministry of Health (MOH) that was characterized as dependent (advocacy, empowerment, delegated) or interdependent (collaborative, cooperative, consultative) was mostly associated with positive outcomes e.g., capacity building; strengthening legislation and stakeholder coordination. Fragmentation (independent engagement) hindered risk communication between public, private, and NGO health programs.

**Conclusion:**

A framework for assessing pharmacogovernance would support policy makers’ evidence-based decision making regarding investments to strengthen capacity for pharmacovigilance and guide policies regarding the state and exogenous actor relationship pertaining to pharmacogovernance. Ideally, dependency on exogenous actors should be reduced while retaining consultative, collaborative, and cooperative engagement when inter-dependency is appropriate. The use of global actors to address Kenya’s pharmacovigilance inadequacies leaves the country vulnerable to 1) ad hoc drug surveillance; 2) pharmacovigilance fragmentation; 3) shifting priorities; and 4) cross purpose interests.

## Background

Pharmacogovernance is essential to policy choices (or lack thereof), infrastructure, institutional authority and resources to address pharmaceutical safety and safeguard public health. It is defined as the manner in which governing structures; policy instruments; institutional authority (e.g., ability to act, implement and enforce norms, policies and processes) and resources are managed to promote societal interests for patient safety and protection from adverse drug reactions (ADRs) [1]. Weak pharmacogovernance negatively affects patient safety because it can lead to a failure to adopt legislation and norms for pharmacovigilance resulting in poor quality medicines in the supply chain. It is costly when limited healthcare resources are wasted on increasing medicines costs; some of which may be unsafe or ineffective [2–5].

In Kenya, the Ministry of Health is responsible for governing the safety of medicines. The Ministry delegates this responsibility specifically to one of its institutions - the Pharmacy and Poisons Board (PPB). The PPB was established by Parliament in 1957 under the Pharmacy and Poisons Act, Chapter 244 [6]. It is a semi-independent regulatory authority [7]. The PPB’s stated mission is to ‘safeguard the health of the public by ensuring that medicines and health products comply with acceptable standards of quality, safety and efficacy’ [8]. PPB’s mission complements the Kenya Health Policy and the Constitution of Kenya which expresses that ‘every person has the right to the highest attainable standard of health’ [Kenya Bill of Rights Article 43 (1)(a)], and ‘consumers have the right (*a*) to goods and services of reasonable quality; (*b*) to the information necessary for them to gain full benefit from goods and services; (*c*) to the protection of their health, safety, and economic interests’ [Bill of Rights Article 46 (1)] [9, 10]. Kenya’s National Drug Policy, Pharmacy and Poisons Act [Rev. 2012], Food, Drug and Chemical Substance Act [Rev. 2012] (FDCSA), and Public Health Act are the key policy and enabling legislation regulating pharmaceuticals in Kenya.

The allocation of financial and human resources needed to support patient safety and protect the public from ADRs is another key component of pharmacogovernance. The PPB can generate revenues to support its activities by collecting fees from pharmaceutical companies for licensing and product registration as permitted by the Pharmacy and Poisons Act. The PPB also receives funding from the national government and ad hoc contributions from international actors such as the European Commission and United States Agency for International Development (USAID) [11, 12]. Human resources are not apportioned equally to PPB’s six Directorates. Staffing is greatest in the Directorates of Business Support (46) and Pharmaceutical Inspectorate, Surveillance, and Enforcement (62). In contrast, fewer staff are allocated to the Directorates of Medicine Information and Pharmacovigilance (5) and Quality Control (2) [11]. Each Directorate is allocated an operating budget by PPB. Some Directorates receive greater funding than others depending on PPB and Ministry of Medical Services priorities. The percentage of PPB’s funding that is allocated to pharmacovigilance is unknown. Funding levels needed to meet PPB’s strategic goals for pharmacovigilance are not reported in the Pharmacy and Poisons Board Strategic Plan 2014–2019 [11].

### Pharmacogovernance and exogenous actors in Kenya

The Kenyan National Pharmaceutical Policy calls for international collaboration and effective partnerships to address pharmaceutical sector issues and to safeguard public health and safety’ [13]. The policy is aligned with Kenya’s national governance that permits the delegation of authority to exogenous (external and non-state) actors to create infrastructure and implement domestic programs that increase national capacity to provide services. They comprise two of the three pillars of the Kenya Health Sector Strategic and Investment Plan (KHSSP) implementation framework: state actors, non-state actors, and external actors [7]. External actors (e.g., bilateral, multilateral, or philanthropic actors), non-state actors (e.g., faith-based organizations, civil society organizations and nongovernmental organizations [NGOs]), international NGOs (INGOs) and not-for-profit NGOs are described in the Kenya Health Sector Strategic and Investment Plan (KHSSP) as ‘crucial partners, both as a financial resource for the health sector and as a way of ensuring programme delivery competencies’ (p.31) to augment limited domestic human resources in the Kenya Health Policy 2012–2030 strategic vision [7, 10].

In 2014, there were more than 7000 NGOs registered in Kenya; most were funded by international donors [14, 15]. NGOs and faith-based organizations provided approximately 14% of health care services in Kenya [16–18]. Other providers of health care services in Kenya are public institutions run by the government (e.g., the Ministry of Health, military, prisons, or state corporations), private facilities, academic institutions and parastatals. The majority of Kenya’s health facilities are public institutions (48%) or private facilities (37%) [17]. Exogenous actors influencing pharmaceutical policy and programs in Kenya include the World Health Organization (WHO), United States Agency for International Development (USAID) and Management Sciences for Health (MSH) [19].

#### Exogenous actors: benefits

The government of Kenya has sought engagement with exogenous actors to address some of the weaknesses of the country’s National Pharmaceutical Policy and PPB’s pharmacogovernance in order to combat deficiencies that leave the country vulnerable to substandard, spurious, falsely labelled, falsified and counterfeit (SSFFC) medicines entering the supply chain. This strategy is a rational one given that dealing with SSFFCs is an issue that goes well beyond one nation’s borders and requires collective action on the part of many stakeholders. For example, in 2011, SSFFC drugs found in Kenya’s supply chain included the oral contraceptive Postinor-2® (levonorgestrol) and 15,000 of batches of the antiretroviral Zidolam-N® (zidovudine) [20]. What is troubling about this example is that the SSFFC antiretroviral medicines that entered the supply chain were not distributed by nefarious groups but rather inadvertently distributed to patients by Médicins Sans Frontières [21].

Kenya has therefore engaged with global actors to increase its capacity to identify SSFFCs, analyze drug safety signals and enhance risk communication [6, 22]. Their resources contributed to advancements in postmarket drug safety by supporting the development of ADR reporting tools (e.g. USAID funding to develop PPB’s online ADR reporting system) and pharmacovigilance training to build capacity for pharmacosurveillance [6]. NGO’s technical support and financial resources enabled active pharmacosurveillance employing cohort event monitoring (CEM) and targeted spontaneous reporting (TSR) (e.g., the Academic Model Providing Access to Healthcare [AMPATH] pharmacosurveillance program at Moi Teaching and Referral Hospital in Eldoret, Kenya). Donor-run health programs have enhanced pharmacovigilance because the programs have pharmacovigilance requirements not mandated by the public sector [22].

Exogenous actors such as the New Partnership for Africa’s Development (NEPAD) African Medicines Regulatory Harmonization (AMRH) Programme have contributed to regulatory governance through the creation of Regional Centres of Regulatory Excellence (RCOREs). In 2014, the Kenya Pharmacy and Poisons Board was designated a RCORE for pharmacovigilance. Kenya is also a member of the East African Community (EAC) which advocates for harmonization and regulatory convergence pertaining to pharmacovigilance [23].

#### Exogenous actors: risks

There is generally good will among state and NGO actors who are perceived as instrumental in implementing policy and programs in Kenya [14]. Olsson, Dodoo and Pal (2015) positively describe numerous examples of global actors’ interventions to catalyze awareness of pharmacovigilance and expand capacity for pharmacovigilance. However, the parallel donor-run health programs that are positively received for mandating pharmacovigilance are also described as negatively contributing to fragmentation in the health system [13]. A key governance challenge, cited in the Sessional Paper for Kenya’s National Pharmaceutical Policy, is overcoming a ‘lack of effective coordination leading to fragmentation and duplication’ of services which is coupled with ‘inadequate technical oversight of private, mission and NGO pharmaceutical service providers’ [13]. Parallel systems for collecting data about ADRs and adverse vaccine reactions coupled with poor data exchange and communication hinder comprehensive pharmacovigilance in many low- and middle income countries [22].

## Theoretical framework

We employ *Network Governance Theory* to explain how and why state and exogenous actors form networks in order to advance postmarket drug safety in Kenya. *Network Governance Theory* proposes that stakeholders may form policy networks to address complex problems that are uncertain, require specialized knowledge, are multi-jurisdictional and have high potential for risk or conflict which members perceive are best solved collectively [24]. *Network Governance Theory* is germane to pharmacogovernance in Kenya where a network of relevant stakeholders contributes to shared policy making. Pharmacogovernance policy and practice are shaped by networks of domestic and global actors who have an influence on the way pharmacovigilance is defined, how challenges are understood and which norms are requisite to address drug safety issues [24, 25]. Many of the exogenous actors that contribute to pharmacogovernance in Kenya are the same non-state and external actors outlined in the KHSSP implementation framework. They contribute to governance through their representation in the Kenya Aid Effectiveness Group (AEG). The AEG is comprised of representatives of the Government of Kenya (GoK) and 17 development partners from 13 countries and the European Commission, the United Nations and the World Bank Group [26]. The AEG serves as a platform for exogenous actors to contribute to governance in Kenya by guiding the joint development GoK-development partner framework for addressing and monitoring issues of mutual accountability [26]. Intergovernmental organizations (IGOs), technical INGOs and philanthropic organizations contributing to pharmacogovernance include: Management Sciences for Health, the World Health Organization, Mission for Essential Drugs, Health Action International- Africa, the New Partnership for Africa’s Development (NEPAD) Regional Centers for Regulatory Excellence and pharmaceutical companies. Their contribution to assessment of Kenya’s pharmaceutical sector influenced the draft *Guidelines for Kenya’s National Pharmacovigilance System*, informed drug safety policies and recommendations for the establishment of training centres for pharmacovigilance governance and advocacy for legislation to advance pharmacovigilance (Table 1) [6, 19, 27].

**Table 1:** Exogenous actors contributing to pharmacogovernance

Exogenous actors	Governance network	Agency	Pharmacogovernance in Kenya
External actors			
Denmark	AEG, SWG	Danish International Development Agency	• Kenya National Pharmaceutical Policy- 2008 [14] • Framework for addressing and monitoring issues of accountability [13]
Germany	AEG	Deutsche Gesellschaft für Technische Zusammenarbeit	• Framework for addressing and monitoring issues of accountability [13]
Japan	AEG, SWG	Japanese International Cooperation Agency	• Corporate governance and technical, financial support • Framework for addressing and monitoring issues of accountability [13]
United Kingdom	AEG	Department for International Development [UK]	• Kenya National Pharmaceutical Policy- 2008 [14] • Framework for addressing and monitoring issues of accountability [13]
United Nations	AEG, SWG	United Nations Children's Fund, United Nations Population Fund	• Corporate governance and technical, financial support [11] • Framework for addressing and monitoring issues of accountability [13]
United States	AEG, SWG	United States Agency for International Development, US President’s Emergency Programme for AIDS Relief, US Pharmacopoeia, Center for Disease Control	• National Pharmacovigilance Guidelines (USP) [6] • Corporate governance and technical, financial support [11] • Framework for addressing and monitoring issues of accountability [13]
European Commission	AEG	Monitoring Medicines Project FP7	• Kenya National Pharmaceutical Policy- 2008 [14] • Normative framework for pharmacosurveillence [44] • Framework for addressing and monitoring issues of accountability [13]
World Bank	AEG	World Bank	• Assessment of the pharmaceutical sector and support for updating policy frameworks [14] • Corporate governance and technical, financial support [11]
Global Fund		Global Fund	• Corporate governance and technical, financial support [11] • Pharmacovigilance norms [17, 45]
New Partnership for Africa’s Development (NEPAD)	AMRH	AMRH	• Regional Centre for Regulatory Excellence in Pharmacovigilance [11, 15]
Management Sciences for Health	PPB and MoH SAGs, County HMT	United States Agency for International Development	• Kenya National Pharmaceutical Policy- 2008 [14] • Kenya National Pharmacovigilance Guidelines- Draft [6]
Non-state actors			
World Health Organization (WHO)	HSWG, PPB and MoH SAGs	World Health Organization (WHO)	• Kenya National Pharmacovigilance Guidelines- Draft [6] • Kenya National Pharmaceutical Policy- 2008 [14] • Assessment of the pharmaceutical sector and support for updating policy frameworks [14] • Pharmacovigilance norms [17]
Uppsala Monitoring Centre (UMC-Sweden); WHO Collaborating Centre for Advocacy and Training in Pharmacovigilance (UMC-Africa)	PPB and MoH SAGs	WHO International Centre for Drug Monitoring	• Kenya National Pharmacovigilance Guidelines- Draft [6]
International NGOs			
Mission for Essential Drugs (MEDS)	PPB and MoH SAGs	MEDS	• Kenya National Pharmacovigilance Guidelines- Draft [6]
Health Action International- Africa (HAI-Africa)	PPB and MoH SAGs	HAI- Africa	• Kenya National Pharmacovigilance Guidelines- Draft [6]
Pharmaceutical Industry	PPB and/or MoH SAGs	Various drug companies	• Policy, law and regulation (QPPV, PSUR) [5, 6]

*(AEG)* Aid Effectiveness Group, *(SWG)* Health Sector Working Groups, *(HMT)* Health Management Team, *(PPB)* Pharmacy and Poisons Board, *(MoH)* Ministry of Health, *(SAGs)* Stakeholder Advisory Groups, *(QPPV)* Qualified Person for Pharmacovigilance, *(PSUR)* Periodic Safety Update Report

## Network actor relationships

Exogenous actors have the potential to continue influencing pharmacogovernance in Kenya given their strong participation in health policy networks. The state-NGO’s relationship however is in a state of flux. The NGO Coordination Act, which was enacted in 1990 and revised in 2012 to coordinate the work of national and international NGOs operating in Kenya, increase oversight, reduce fragmentation in the healthcare system and provide policy guidelines regarding harmonization of activities for the national development plan for Kenya, through a NGO Coordination Board, was repealed and replaced by the Public Benefit Organization (PBO) Act, passed January, 2013 [28]. The PBO Act provides clear definitions for what is and is not a PBO and what type of activities that PBOs can and cannot engage in. PBO activities include: enhancing or promoting the economic, environmental, social or cultural development or protecting the environment or lobbying or advocating on issues of general public interest or the interest or well-being of the general public or a category of individuals or organizations [29]. While the country continues to recognize *‘the importance of mutual co-existence and the need to work together [with NGOs]’* [29], editorials have suggested that the Kenyan government has been pushing forward constitutional amendments such as the new PBO Act, to *‘shrink the political and legal space in which they operate’* [30]*.*

A framework for understanding the state and exogenous actor relationships pertaining to pharmacogovernance is critical, given Kenya’s past and current dependence on exogenous actors as implementing partners. We thus investigated the relationships between specific modes of engagement amongst exogenous and domestic actors at the national and subnational level to identify the positive or negative effect on pharmacogovernance and pharmacovigilance. The research question addressed through this study is: Which pattern(s) of engagement among exogenous actors, the Kenya Pharmacy and Poisons Board and county actors enable or hinder pharmacogovernance and pharmacovigilance?

We analyzed dependent, independent, and interdependent patterns of engagement amongst state and exogenous in Kenya. We defined dependency as *relying on others for financial or other support*. Dependency is an asymmetrical relationship whereby global and domestic actors with resources and political power are able to influence policies and processes in tandem with or in conflict with the interests of lessor resourced local groups [31]. Independence was defined as *not depending on another's authority or resources for support*. In interdependent modes of engagement *each partner benefits by reliance on the other*. Interdependence between actors may be equal and mutually dependent (symmetrical) or asymmetrical [32]. The actions or national policies of one partner are perceived to have a direct effect on the other members, although the policies may benefit partners disproportionately [32]. Kenya is a robust example to examine because of the numerous internationally funded nongovernmental organizations (NGOs) operating in the country and their high level of integration into Kenya’s governance [14].

Our paper is organized as follows. First, we provide background regarding pharmacogovernance and global actors in Kenya. Second, we describe our research methods. Third, we analyze exogenous actors’ motivation for engaging with state actors to advance pharmacovigilance and pharmacogovernance. Fourth, state and exogenous actors’ perceptions of pharmacogovernance and drug safety are analyzed. Finally, the patterns of engagement between state and exogenous actors are characterized and analyzed.

## Methods

Key informant interviews and a documentary analysis were conducted to investigate the patterns of engagement between state and exogenous actors affecting pharmacogovernance and pharmacovigilance in Kenya. Purposive and reputational sampling strategies were employed to select participants able to provide rich data about pharmacovigilance systems in Kenya. The participants were identified via a ‘snow-balling’ process. Thirteen key informants were interviewed which represented the calculated quota sample. Quota sampling is a non-probablistic sampling method used to determine the *minimum* representative sample size [33]. In this case study, a quota sample representing location (rural, urban), pharmacovigilance resources (high resourced, low resourced) and sector representation (regulatory), government (national, external [IGO]), pharmaceutical manufacturer, non-state actor, non-governmental organization (national, international) was interviewed. The key informants included representatives of the Kenya medicines regulatory authority (Pharmacy and Poisons Board), multinational and domestic pharmaceutical corporations, IGOs/INGOs and pharmacy and Kenyan government representatives from Turkana County, Uasin Gishu County, Mombasa County and Kwale County. The county key informants that were interviewed represented a convenience sample of information-rich individuals from urban and rural counties. The sample was selected to characterize regional differences in pharmacogovernance and pharmacovigilance in Kenya. See Table 2.

**Table 2:** Characterization of key informants interviewed

#Key Informants Interviewed	Sector	Region
2	Regulatory	National
4	Health/Pharmacy Administrators	County
2	Multinational Pharmaceutical Companies	Global
1	Domestic Pharmaceutical Company	National
4	IGO/INGO	Global

Interviews were conducted in person or by SKYPE between March 2014 and January 2015 following ethics approval from the University of Toronto, Canada and Moi University, Kenya. Informed consent including consent to publish the information they provided was obtained from study participants. Interviews were conducted until saturation of themes was reached. All interviews were audio recorded and transcribed verbatim.

Interview data and publicly available government documents (e.g., the Kenya constitution (2010), the Pharmacy and Poisons Act and the NGO Coordination Act), peer reviewed and grey literature and newspapers archives were read iteratively. Data were coded into key themes employing an open coding process using Atlas ti v. 7.5.9 (2015) qualitative software. Data coding and re-coding was an ongoing process as new and more refined patterns of engagement between exogenous and state actors emerged [34]. A codebook with operational definitions was created to maintain consistency in the coding process (Appendix A).

A semantic analysis of key informant transcripts was also conducted. A semantic analysis investigates how words are used in context and their relationship to one another [34]. Words used by key informants to describe their perceptions of pharmacovigilance priorities at federal, county, and corporate levels; governance related to the priority setting process; the nature of interactions among global, national, county and municipal actors pertaining to pharmacovigilance were analyzed. An analysis of linguistic connectors used by key informants, provided insight into their perspectives of causality (*‘because’)*, conditional relations (*‘since’)*, contingent relations (‘*if-then, rather than’*; ‘*as soon as’)* and temporal relations (‘*before, now’)* related to how, when and why state and exogenous actors interacted pertaining to pharmacogovernance [34].

## Results

### Exogenous actors’ motivation for advancing pharmacovigilance in Kenya

The semantic analysis of key informants interviewed suggested that exogenous actors have begun to prioritize pharmacovigilance. According to one IGO/INGO interviewed, *“there are very many good reasons to expand and extend the coverage of pharmacovigilance because the chance of finding rare adverse reactions is directly proportional to the population exposed and the danger that is being gathered. In terms of the variability of the problems, they will look very different in different populations and different cultural situations* (IGO/INGO-2). For this reason, they have funded active surveillance studies as well as pharmacovigilance training. As a member of the Uppsala Monitoring Centre, PPB has received access to data analyses of case reports and the Vigibase® data management system. Although historically their priorities for commodities management have focused on access rather than pharmacovigilance, the global actors that were interviewed actively advocated for pharmacovigilance within the programs that they managed because *“…you can see basically that’s commodities management. Pharmacovigilance is just one [part] of it. I talk passionately when I advocate for pharmacovigilance. I say that we have to match ACCESS with SAFETY”* (IGO/INGO-5).

International donors’ priorities for pharmacovigilance have influenced domestic priorities at the regulatory authority and county level. Input from the WHO contributed to the expansion of the Pharmacy and Poisons Board priorities to include medicines safety [13].

*“There was a time when we [only] wanted medicines to be available. That was the biggest want. But now we want to go past the availability debate to the safety of these drugs.”-* PPB-1

*“There could be [a relationship between priorities for pharmacovigilance in the county and global actors] because many times when we conduct this postmarket surveillance we are funded. So there could be a connection in that these donors might be funding it.” -* County-4

Exogenous actors’ engagement with the Government and the Pharmacy and Poisons Board has focused on strengthening pharmacogovernance in Kenya to enhance postmarket drug safety. An Indicator-based Pharmacovigilance Assessment Tool (IPAT) was developed by USAID/MSH [35] and in 2015, the WHO published an up-to-date comprehensive tool for assessing pharmacovigilance systems [36]. In addition to guidelines for pharmacovigilance system assessment [37], they have supported the establishment of pharmacovigilance centres in Kenya, and other countries in Africa; disseminating their policy norms through them.

“*Our work plan is also strengthening those that have come on board or there is no point in being on board. And, helping them with systems issues like policies… So I came to them championing partnerships where they are drumming up support from those around them.”* -IGO/INGO-3

### State and exogenous actors’ perceptions of pharmacogovernance and drug safety

Pharmacogovernance affects governing structures; institutional authority; implementation of law and policy; enforcement of laws, norms, policies and processes; and resources to advance drug safety.

The results of our semantic analysis revealed that key informants’ perceptions of the relationship between governance and pharmacovigilance converged regarding the desire for harmonization of policy and laws for drug approval and postmarket requirements. Key informants’ perceptions regarding other facets of pharmacogovernance varied according to type of actor interviewed. International NGOs used the most words to describe governance; reflecting their perception of its importance. The importance of good governance is also widely disseminated in their literature [19, 38–40]. A code of ethics to prevent corruption, good manufacturing processes, spontaneous ADR reporting and sentinel reporting sites are described as requisites of good governance pertaining to pharmaceuticals [37, 38, 40]. Words that were used by IGO/INGOs in their response to interview questions about governance included accountability, transparency, budgets and funding, autonomy, leadership, contracts, rules and directives.

The words that PPB key informants used in their response to interview questions related to governance reflects their perception that achieving their mission ‘to safeguard the health of the public by ensuring that medicines and health products comply with acceptable standards of quality, safety and efficacy’ is affected by the National Pharmaceutical Policy, institutional authority and capacity. Kenya’s pharmaceutical sector has been characterized as having a weak infrastructure, conflicting laws and weak enforcement of laws governing pharmaceuticals [13, 19]. Enforcement of pharmacovigilance has been challenged because *“the law is not explicit on pharmacovigilance issues”* (PPB 02). The words that representatives of PPB used related to governance included constitution, policy, budget and revenue. The emphasis on resources reflects the perception that current budget and staffing for pharmacovigilance is insufficient to cover pharmacovigilance, postmarket surveillance, and medicines information for 2 million Kenyans (PPB-01).

Pharmaceutical representatives interviewed held divergent perceptions of their companies’ responsibility for pharmacogovernance. In regards to accountability for drug safety, all of the pharmaceutical representatives that were interviewed perceived that their primary responsibility was to adhere to corporate pharmacovigilance policies. They perceived it was in their companies’ interest to influence PPB norms and policies pertaining to ADR reporting. The words used by pharmaceutical companies to describe governance emphasized policy and standard operating procedures*.*

*“So with the corporate governance we’ve got policies and procedures, worldwide policy and procedures, we’ve got SOPs that are global.”-* Pharma-1

Only one of the pharmaceutical company representatives interviewed highlighted the importance of a corporate governance policy for company-wide participation in pharmacovigilance.

### Patterns of interactions among state, non-state, and external actors’ influencing pharmacogovernance and pharmacovigilance

We analyzed the patterns of engagement between exogenous and state actors and found examples of dependent (advocacy, delegation, empowerment and hierarchy), independent (autonomy and fragmentation) and interdependent (collaboration, cooperation, and consultation) patterns of engagement. Dependent and interdependent patterns of engagement were found to be associated with strengthening pharmacovigilance through capacity building; pharmacovigilance priority setting; advocacy for policy, law, or regulation; stakeholder coordination; and local empowerment for pharmacovigilance. Delegation was associated with strengthening pharmacovigilance with respect to exogenous actors’ aim to develop Pharmacy and Poisons Board into a regional actor for pharmacovigilance for the purpose of transferring to Kenya the responsibility for building regional capacity. The effect of specific modes of engagement on pharmacogovernance is shown in Table 3.

**Table 3:** The modes of engagement between domestic and exogenous actors and the effect on pharmacogovernance

Pharmacogovernance domains	Modes of Engagement							
	Advocacy	Autonomy	Collaborative	Consult -ative	Cooper -ative	Delegated	Empowerment	Fragmented
Policy, law, regulation (Governing structures, norms, policy instruments, practices, institutional authority)	+/-	+/-	+	+	+	+		
Accountability & Transparency	+			+				
Participation & Representation				+/-				
Equity & Inclusiveness (Distribution of resources for pharmacovigilance)	+		+					
Ethics (Policies to safeguard patient interests)	+/-							
Effectiveness & Efficiency (System integration & communication)								-
Responsiveness (Risk communication)				+			+	
Intelligence & Information (e-reporting technology, risk communication)	+		+			-		-
Stakeholder Coordination (Pooled resources, network mobilization, communication network)	+		+		+			_

Enable pharmacovigilance (+), Hinder pharmacovigilance (-)

## Interactions strengthening pharmacogovernance

Exogenous actors have both catalyzed and strengthened some of the proposed changes to pharmaceutical policies, laws and regulations in Kenya [6, 10, 19]. They engaged in advocacy with government, Pharmacy and Poisons Board and other non-state stakeholders to shape PPB and Ministry of Health policy preferences regarding the Pharmacy and Poisons Act Amendment and adoption of a National Pharmacovigilance System. They have engaged in consultative interactions for the purpose of sharing information about pending legislation and changes in existing legislation.

*“[We] liaise with government to get to know what needs to be done, what legislation, what laws need to be put in place, what reviews need to be made, what needs to be taken to Parliament just to get the legislation in law, and all that. You know, negotiating at the government level to make sure the right laws get passed.”* - IGO/INGO-1

However, one non-state actor expressed frustration with the pace of change suggesting that it had hindered pharmacovigilance *“because once it goes through Cabinet they change many things so there are many things that I have had to pull, because of these new changes as far as governance goes to tweak implementation. I can’t implement it under the previous budget because of new laws!” (IGO/NGO-5)*

The IGO/INGOs key informants advocated for greater accountability by pharmaceutical manufacturers because, *“industry is manufacturing medicines and releasing them out to the market and thinks once it is sold…to them it is done”* (IGO/INGO-1)*.* The need for greater industry accountability was echoed by a pharmaceutical industry key informant who reported that companies that invested in pharmacovigilance were competing against companies that did not; suggesting that self-regulation was ineffective.

Despite Kenya’s adoption of global actors’ norms for pharmacovigilance, key informants that were interviewed asserted that the basis for engagement between state and exogenous actors in Kenya was autonomy. As such, they claimed that they merely ‘suggested’ policy, laws, regulations and norms for adoption. They respected that “*[low- and middle-income countries (LMICs)] know where they are going*” (IGO/INGO-5). Rather than drive a specific agenda, key informants claimed that they responded to requests for support by local/national governments when they had overlapping interests.

*“We are aware that individual countries or regions need to develop, like European Commission did back when the European Union did not have an EU-legislation on pharmacovigilance. We also are aware that this has to evolve.*” – IGO/INGO-3

Exogenous actors’ engagement with the PPB was largely consultative regarding setting pharmacovigilance priorities “*to determine what our focus areas really should be and once determined, support aspects of quality assurance of medicines issues and aspects of ensuring that patient safety is optimal”* (IGO/NGO-1). Data anayzed from actors at the policy level (government), implementation level (health facilities), oversight level and the private sector showed that, *“agreed milestones and timelines of giving this support”* were established as a consequence of the consultations (IGO/NGO-1). One key informant stated that when meeting with the County Health Executive, *“we were looking out for all of these things together, the commitment they are making, and listening to the plans that they have and telling them what we are looking out for and what we plan to do in the future.”* (IGO/NGO-1)

Kenya PPB, IGO and INGO key informants’ policy preferences for pharmacovigilance are compared in Table 4.

**Table 4:** Comparison of pharmacovigilance priorities between state and exogenous actors

PPB	County	Generic pharma	Multinational corporation	INGO (non-state)	IGO (external actors)
• Drug safety in addition to availability • Increase ADR reporting (public & health workers) • Poor quality medicines reporting • Active surveillance • Ensure quality of products in the market • Harmonization of PV system • Pharmaco-surveillance of commonly used drugs and drugs used in public programs (e.g., TB, and malaria). • Increase awareness of PV • Capacity building • PV Legislation (No explicit PV laws) • Kenya as a leader in patient safety-a Centre of Excellence • Facilitate ease of reporting • Take action on ADR reports • Introduce industry reporting	• Drug safety in addition to access • Detect and report ADRs • Detect and report poor quality medicines • Monitoring quality of medicines • Strengthen PV systems • Pharmaco-surveillance of drugs targeted by public programs (TB, family planning, and malaria; high morbidity, commonly used) • Quality testing of drugs entering through ports • Health facilities know proper storage conditions for medicines	• Substandard, fake, and counterfeit drugs	• Harmonization of reporting requirements • Training for all employees to increase awareness of PV • PV as a clearly defined role • Maintain compliance with national PV requirements • Spontaneous reporting only- no mandatory reporting	• Drug safety in addition to access • PV Systems strengthening • Capacity building to improve the quantity and quality of ADR reports • PV Legislation (e.g., qualified PV personnel in each drug company) • Synchronize priority areas with PPB and other IGO/INGOs • Systems surveillance approach to PV • Establish PV systems • Adoption of a National Pharmacovigilance Policy	• Increase policy makers and donors awareness of the connection between access and PV Health systems strengthening • Active surveillance • Ensure quality of products procured • Harmonization of PV systems and reporting tools, nationally • Capacity building to improve the quantity and quality of information that supports decision making. • Negotiate with government to achieve an appropriate legal framework for PV • Support for pharmaceutical policies and laws • All countries have PV systems. • Global populations are covered by PV activities. • Participation in UMC is expanded to increase the chance of finding rare adverse reactions • Redefine the focus of PV to include old issues with older drugs (Evergreening)

Some exogenous actors have engaged in collaborative interactions with the counties to provide resources to support greater ADR reporting and to facilitate dissemination of ADR reports submitted.

*“We normally support that because health care workers report and the reports are laying [around] in the facilities because they don’t necessarily have a system of how they are getting to the Pharmacy and Poisons Board. So that is both for ADRs and reports for the whole country not just in certain counties.”* - IGO/INGO-5

Several key informants reported that IGO/INGOs’ advocacy in Kenya was part of a broader agenda, to strengthen pharmacovigilance regionally. A network of pharmacovigilantes that have been trained in Kenya have been delegated the responsibility to educate Kenya’s neighbours, build local capacity for pharmacovigilance and advocate for regional strengthening of pharmacovigilance systems. PPB will be able to implement this agenda in their capacity as a Regional Centre of Regulatory Excellence (RCORE) for pharmacovigilance.

*“Officers from PPB are part of our outreach strategy, part of Pharmacovigilantes Sans Frontiers… and it’s working. The more you can influence big countries the more you use them to influence those around them.”* - IGO/INGO-3

## Interactions hindering pharmacogovernance

Certain interactions negatively influenced pharmacogovernance, specifically policies for ADR reporting and corporate accountability for pharmacovigilance. One key informant reported that the PPB sought industry input regarding policy changes and was responsive to industry requests. Industry input was sought on the 2014 draft legislation to require each pharmaceutical company to employ a qualified person for pharmacovigilance (QPPV). Another informant reported that PPB *“obviously wants to engage with industry, wants collaboration, and wants our input”* (Pharma-1). The key informant reported further that PPB had been responsive to previous requests including a request to modify the schedule for completing the Periodic Safety Update Report (PSUR) to harmonize with the reporting schedule for regulatory authorities in the European Union.

**Fig. 1:**
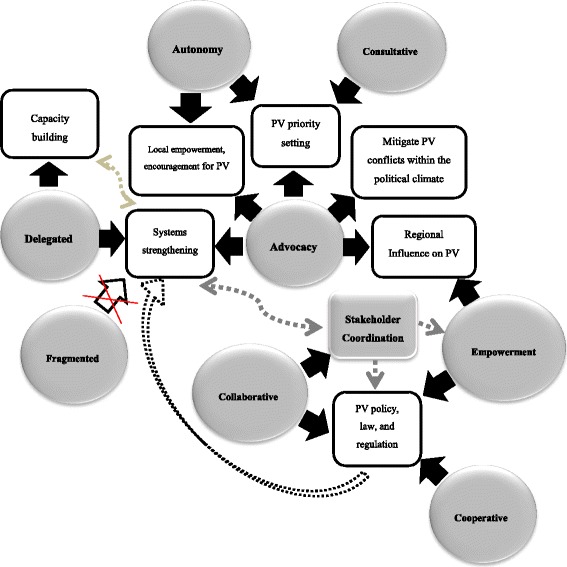
Patterns of interactions between domestic and exogenous actors affecting pharmacovigilance

The patterns of engagement between exogenous and domestic actors and their effect on the pharmacogovernance domains are shown in Fig. 1.

## Interactions strengthening pharmacovigilance

Non-state and external actors used the power of their existing networks to advocate for the creation of new networks for the purpose of mobilizing resources to strengthen pharmacovigilance and active pharmacosurveillance. Pooling resources expanded capacity for pharmacovigilance thus creating a better value for limited donor dollars. The European Commission, WHO and UMC worked with PPB to implement Cohort Event Monitoring, *“they provided seed money and they supported us to develop the clinical trials registry in collaboration with the Kenya Medical Institute”* (PPB-2).

*“We’ll pool [resources]. Like that Cohort Event Monitoring program, the HIV and us…We work together. They have put in some support or even technical assistance for that. During the implementation of that program we draw on that money to support it. We also use the money from there to train the sites”. -* IGO/INGO-5

IGO/INGO advocacy contributed to developing a cadre of advocates for pharmacovigilance whom they nurtured and exposed to pharmacovigilance best practices and training to build confidence and competence. The term *pharmacovigilantes* was used to describe individuals who advocated for pharmacovigilance. The manner in which global actors courted and nurtured the pharmacovigilantes is exemplified by the following quote:

*“In Kenya in particular, one of the key people we identified very early on in Pharmacovigilantes Sans Frontiers, we courted him and nurtured him… we provided exposure to best practices outside and in Kenya starting from way back in 2004 and started sowing the seeds. So through our direct efforts there is a pharmacovigilance centre and [it] became affiliated with the WHO program. And now they’re one of the leading countries in Africa.”-* INGO/NGO-2

## Interactions hindering pharmacovigilance

Some pharmaceutical companies’ actions to influence Kenya’s draft legislation requiring a qualified person for pharmacovigilance (QPPV) may negatively impact both pharmacogovernance and pharmacovigilance. The proposed legislation would require each pharmaceutical company operating in Kenya to employ pharmacovigilance personnel. In the absence of QPPV legislation, the multinational corporations (MNCs) interviewed had assigned a single individual to oversee more than 25 countries. One key informant was responsible for overseeing 42 African countries. Corporate decisions to appoint a regional head for pharmacovigilance rather than a country head and to oppose QPPV legislation impedes patient safety and therefore contradicts beneficence.

*“It’s impossible for a pharmaceutical company to have a pharmacovigilance person, totally responsible for pharmacovigilance in each country. So, they [PPB] have [proposed] a stricter guideline which I have written back and said we can’t do that.”*- Pharma-1

**Fig. 2:**
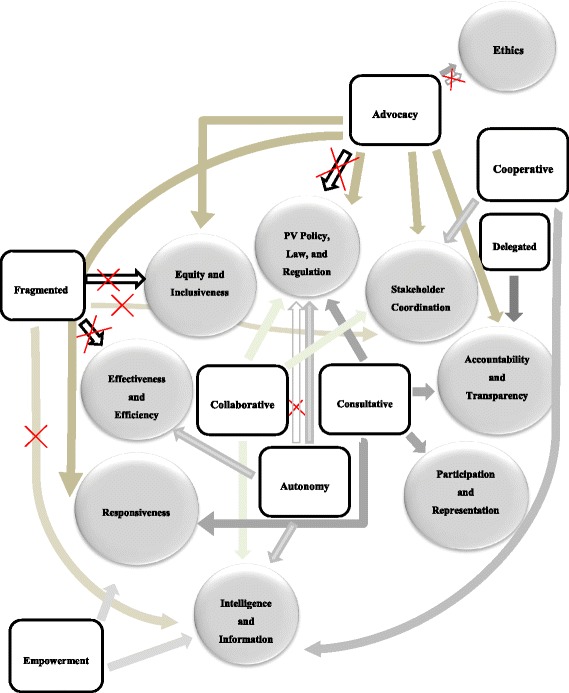
Patterns of interactions between domestic and exogenous actors affecting pharmacogovernance

The relationship between pharmacovigilance and patterns of engagement between state and exogenous actors is shown in Fig. 2.

## Discussion

Pharmacogovernance and pharmacovigilance in Kenya mirror other low- and middle income countries [1, 20, 22]. Many Sub-Saharan Africa countries lack a national pharmacovigilance system, have limited infrastructure, human resources, training and capacity to detect and analyze drug safety signals [20, 22]. A survey of Sub-Saharan Africa countries found that fewer than one third had laws and regulations to mandate pharmacovigilance activities [20]. Pharmaceutical safety issues are not confined to regional borders. Risks related to pharmaceutical consumption are multi-jurisdictional because medicines are produced and sold in a global marketplace [13]. Medicines manufactured in Kenya are marketed to other countries in the East Africa Community and medicines manufactured by multinational corporations are available for sale in Kenya. *Network Governance Theory* suggests that under such conditions policy actors will be motivated to come together in order to contribute to shaping governance.

The research findings illuminate how Kenya’s governance, that permits delegation of authority to non-state implementing partners, creates space for exogenous actors to influence normative policy, policy instruments and practices that affect pharmacogovernance and pharmacovigilance. Brass (2012), found that NGOs in general are well integrated into Kenya’s governance and the line between government policy makers and NGO implementing partners is blurred.

Our semantic analyses showed that exogenous and domestic actors’ perceptions of governance reflected their divergent interests in shaping the direction of pharmaceutical policy. Whereas IGO/INGOs linked accountability with pharmacogovernance, and described their actions to contribute to shared governance as a mechanism to advance pharmacovigilance globally, some county actors and pharmaceutical firms (domestic and MNC) perceived accountability as the responsibility of others, such as the PPB. This finding is significant in terms of drug safety and suggests that stronger pharmacogovernance between levels of government and among domestic and exogenous actors is needed. Despite differences in the words attributed to governance expressed by sector representatives, our finding that there was shared interest in pharmacogovernance related to shaping rule of law and harmonization of policies among IGO/INGOs, PPB and pharmaceutical firms interviewed is important. The implication of this finding is that regulatory authorities, pharmaceutical firms and global actors can converge around a common solution (e.g., strengthening regulatory governance for medicines safety) despite divergent problem definition when interdependency is perceived to be beneficial.

All of the key informants interviewed perceived engagement among state, non-state or external actors was generally positive; likely because exogenous actors’ priorities for pharmacovigilance were adopted in tandem with domestic interests. Moreover, the Pharmacy and Poisons Board exercised autonomy over its choice to adapt or adopt suggested policy norms. As an example, in designing its e-reporting system, PPB adapted WHO norms for pharmacosurveillance by adding a feature for reporting suspected poor quality medicine. PPB signaled its priority to expand the definition of pharmacovigilance beyond ADRs.

We found that in Kenya, pharmacogovernance and pharmacovigilance are strengthened by fostering interdependent engagement among county, national and exogenous actors. Specifically, engagement among the Pharmacy and Poisons Board, Ministry of Health and exogenous actors based on collaboration and advocacy favoured resource allocation by exogenous actors to support pharmacovigilance and reduce drug safety risk. Through engagement characterized as advocacy, governance networks comprised of domestic and exogenous actors are providing support for expansion of PPB’s regulatory authority and amendments to the Food, Drug and Chemical Substance Act and Pharmacy and Poisons Act in order to strengthen pharmacogovernance. Neither of the Acts included language on pharmacovigilance when first passed.

The state-exogenous actors’ relationship regarding the pharmacogovernance domain *‘Policy, Law, and Regulation’* was mostly positive, however this study found efforts by some exogenous actors to limit regulatory reforms. Pharmaceutical industry consultations with PPB, that aimed to discourage adoption of requirements for a QPPV on the grounds that the policy was unnecessary and too costly, impeded pharmacovigilance.

We argue that pharmacovigilance may also be hindered by exogenous actors’ policy preferences for the harmonization of regulatory requirements for drug registration because the aim of policies promoted by the AMRH and the EAC is to reduce trade barriers rather than pharmacovigilance [7, 41]. Although this study found convergence in key informants’ perceptions of regulatory harmonization, regardless of the sector they represented, the benefits of regulatory harmonization have been contested in the literature due to the trend to harmonize to the lowest standard rather than raise regulatory requirements, even in high income countries [42, 43].

Some of the deficits that were found in the pharmacogovernance were not overcome by state-exogenous actors’ engagement. We did not find evidence that exogenous actors influenced legislation to address the lack of resources for pharmacovigilance. However, we found strong engagement among exogenous actors that had a positive effect on mobilizing donor resources for pharmacovigilance. Interdependent and dependent engagement led to resource allocation for active pharmacosurveillance (e.g., CEM and TSR) as well as innovations in data collection and risk communication (e.g., e-reporting and mobile phone apps). Donor support for pharmacovigilance was commonly cited as critical by key informants because resources available for pharmacovigilance are limited in Kenya and other LMICs [20, 44, 45]. Exogenous actors including USAID, MSH, WHO, Global Fund, and the Bill and Melinda Gates Foundation have recommended multi-sectoral engagement as a mechanism to expand resources for pharmacovigilance in resource-limited settings. Stakeholder coordination was likely strong because a sector-wide approach to development has been widely promoted by the neoliberal agenda since early 2000 [46]. We did not investigate whether certain global actors had more influence on pharmacogovernance than others however, the literature suggests that technical and financial support from transnational policy actors is an incentive for the uptake of policy ideas in under-resourced LMI countries [47]. Thus, exogenous actors that offer financial and technical resources may have the greatest influence on pharmacogovernance in Kenya.

## Conclusions

The research advances our understanding of the patterns of engagement between state and non-state actors pertaining to pharmacogovernance and pharmacovigilance. The literature on *Network Governance Theory* and risk governance posits that responsibility for managing risk should be shared between state and non-state actors [48, 49]. Our finding that domestic and exogenous actors contributed to pharmacogovernance through positive interdependent engagement is consistent with *Network Governance Theory,* which advances that multiple stakeholders’ varied perspectives expands the characterization of risk and the solutions that are considered. The research may be transferable particularly in regards to findings that indicate that the global policy community can support domestic policy actors’ efforts to strengthen pharmacovigilance in Sub-Saharan Africa through participation in governance networks. A global funding model could further advance national pharmacovigilance if funding is targeted to strengthen pharmacogovernance through the development of a national pharmacovigilance system; resource distribution for equitable pharmacosurveillance and the development of a nationwide risk communication network for rapid dissemination of drug safety information.

A framework for assessing pharmacogovernance would support policy makers’ evidence-based decision making regarding investments to strengthen capacity for pharmacovigilance; important when resources are limited [1, 50]. It would also support policy to address inequities in pharmacovigilance particularly in rural geographic regions that are disproportionately disadvantaged; having fewer hospitals and pharmacosurveillance units than urban centres [1, 22, 50].

The research results suggest that caution is warranted regarding the sole use of exogenous actors to fill a deficit in capacity for pharmacovigilance. It leaves Kenya vulnerable to: 1) a fragmented pharmacovigilance system; 2) ad hoc, drug specific pharmacosurveillance; 3) cross purpose interests; and 4) exogenous actors’ shifting priorities. For although exogenous actors are likely to continue to advocate for pharmacovigilance while interests align, Kenya is already experiencing the effect of shifting priorities that have reduced donor funding. Whereas key informants interviewed recognized Kenya’s autonomy to adopt global actors’ norms, less consideration was given to donors’ autonomy to determine to whom to provide their resources and support. Therefore, the research suggests that the strengthening pharmacogovernance amongst national and subnational government levels will best advance postmarket drug safety in low- and middle income countries.

## Limitations and future research

The key informants interviewed for this study represent an information-rich sample of policy makers in IGO/INGOs, government and pharmaceutical firms in Kenya. The data collected represent a range of views pertaining to state and exogenous actor engagement that is limited to pharmacogovernance. We were unable to interview representatives from the faith-based sector. Although data provided in interviews about this sector were reported in the literature reviewed and the documents analyzed, additional studies are suggested to capture the perspectives of representatives from the faith-based sector. Additional studies are needed to evaluate whether the study findings are transferable to other sectors where state/non-state governance networks operate.

## Appendix A

**Table 5:** Patterns of interactions and pharmacogovernance codebook

Patterns of interactions		
Code	Definition	Example
Autonomy	Self-governing or acting independently.	*So that’s the thing with devolution. You can make the decision. You don’t have to wait for somebody to tell you what you do if this or that.*
Advocacy	Motivates change in policies, priorities and/or resource allocation to advance pharmacovigilance	*The country can identify it [pharmacovigilance] as a priority but if it had not been an important focus area from Washington, then it becomes difficult for the local region to get sufficient resources for it.*
Collaborative	Formal or non-structured partnerships for the joint development of programs, policies, and pharmacovigilance resources that strengthen pharmacovigilance.	*[We developed a] Master’s in pharmacy, pharmacoepi, and pharmacovigilance. And we collaborated with the University of Nairobi and the University of Washington. I got it up and running now.*
Consultative	Engages external advisors in pharmacovigilance decision-making.	*So you want to work closely with the County Directors of Health, the County pharmacists so that you can get more information from them. And share with them what you are seeing in other counties because they will only see what is available in the county.* *[T]hey send you their comments. …Cause we cannot do a guideline without the input from the stakeholders. We even require that in our constitution. Anything that is going to affect your stakeholders, it is important that you get input from them.*
Cooperative	Agreement made between federal, state, county, or global actors to work together or jointly support a specific issue.	*[B]ilateral cooperation is agreed among the countries. We may or may not take part. We may suggest some field for cooperation among two countries.*
Delegate	Give oversight of pharmacovigilance activities to another entity or group (e.g., national regulatory authority to county/state dept. of health; MoH to NGO; nurses to pharmacists).	*The staff in the county, they’re mostly drug inspectors; there are no pharmacovigilance case [workers]. So in case the [regulatory authority] wants to conduct pharmacovigilance they will contact me to coordinate the activities in Coast region.*
Fragmented	Interrelated PV responsibilities and overlapping functions with limited integration of PV activities and accountability.	*Yeah, It’s quite complicated for faith-based organizations, at least for the Catholic church. They have their own system.*
Hierarchical	Organized according to a series of levels with different importance or status, each subordinate to the one above it.	*[B]ecause as a global group I don’t report here. My director is based in headquarters. I have a dotted line to the country manager but my group reports directly to headquarters.*
